# Evidence for the continued presence in New Zealand of *Homotrysis macleayi* (Borchmann, 1909) (Coleoptera: Tenebrionidae: Alleculinae)

**DOI:** 10.3897/BDJ.2.e1054

**Published:** 2014-02-04

**Authors:** Stephen E. Thorpe

**Affiliations:** † School of Biological Sciences (Tamaki Campus), University of Auckland, Auckland, New Zealand

**Keywords:** *Homotrysis macleayi*, NZOR, Auckland, New Zealand, Australia, faunistics, data, evidence

## Abstract

The first detailed specimen records are presented for the Australian beetle *Homotrysis macleayi* (Borchmann, 1909) in New Zealand. Evaluation of this evidence clearly indicates that the species is fully established in the wild in New Zealand. It is therefore recommended that the species be added to the New Zealand Organisms Register (NZOR), as exotic and present in the wild. Some general comments are offered on the importance of data and evidence in faunistics.

## Introduction

In 2004, I collected what is probably the first New Zealand specimen of the Australian beetle *Homotrysis macleayi* (Borchmann, 1909). Although I immediately recognised it as a species of alleculine tenebrionid unknown in New Zealand, it was not identified until I found others in 2012. These were identified as *Homotrysis macleayi* by Australian tenebrionid expert Dr. Eric Matthews (South Australian Museum). The species was validated new to N.Z., based on this material identified by Matthews, by Ministry for Primary Industries 2013. Only scant details were published by MPI (i.e. insect, *Homotrysis macleayi* (tenebrionid beetle), *Acacia* sp. (wattle), Auckland, General Surveillance). Nothing more has been published regarding the presence of this beetle in New Zealand. There is currently no record of it on the New Zealand Organisms Register (NZOR). It is therefore somewhat unclear what the status is of the species in New Zealand. Is it a permanently established member of the New Zealand fauna? Faunistics is the study of the presence/absence of species in a given area, such as New Zealand. Since we are scientists, and not stamp collectors, the presence of a given species in a given area should be stated with specification of the associated evidence for its presence. Presence/absence can change over time. Presence could be based on a single, possibly mislabelled and/or misidentified specimen, or by many independent specimen records taken over a long period of time. It is important to specify, but it is not often done. Similarly, presence could be based on a specimen or specimens representing only post border interceptions, without a breeding resident population. Only the systematic accumulation of data can establish the facts.

## Taxon treatments

### Homotrysis macleayi

(Borchmann, 1909)

https://species.wikimedia.org/wiki/Homotrysis_macleayi

Allecula macleayi Borchmann, 1909 (original combination)
Allecula flavicornis Macleay, 1887 (objective synonym)

#### Materials

- **Type status:** Other material. **Occurrence:** recordedBy: S.E. Thorpe; individualCount: 1; **Taxon:** scientificName: *Homotrysis macleayi* (Borchmann, 1909); **Location:** country: New Zealand; stateProvince: Auckland; verbatimLocality: Auckland Domain; verbatimLatitude: 36.86385S; verbatimLongitude: 174.77501E; **Identification:** identifiedBy: Stephen E. Thorpe; **Event:** samplingProtocol: On trunk of Eucalyptus tree at night; eventDate: 2004-04-28; **Record Level:** institutionCode: Auckland Museum; collectionCode: AMNZ57969
- **Type status:** Other material. **Occurrence:** recordedBy: S.E. Thorpe; individualCount: 3; **Taxon:** scientificName: *Homotrysis macleayi* (Borchmann, 1909); **Location:** country: New Zealand; stateProvince: Auckland; verbatimLocality: Tamaki Campus (East), suburb of Saint Johns, Auckland; verbatimLatitude: 36.88615S; verbatimLongitude: 174.85258E; **Identification:** identifiedBy: Eric G. Matthews; **Event:** samplingProtocol: Under loose bark of chopped up wattle tree (possibly Paraserianthes lophantha); eventDate: 2012-03-08; **Record Level:** institutionCode: Auckland Museum; collectionCode: AMNZ86134 (1 specimen)
- **Type status:** Other material. **Occurrence:** recordedBy: S.E. Thorpe; individualCount: 1; **Taxon:** scientificName: *Homotrysis macleayi* (Borchmann, 1909); **Location:** country: New Zealand; stateProvince: Auckland; verbatimLocality: Tamaki Campus (East), suburb of Saint Johns, Auckland; verbatimLatitude: 36.88216S; verbatimLongitude: 174.85331E; **Identification:** identifiedBy: Stephen E. Thorpe; **Event:** samplingProtocol: Under bark of Pittosporum eugenioides stump, at edge of carpark.; eventDate: 2013-05-05; **Record Level:** institutionCode: Auckland Museum; collectionCode: AMNZ87636
- **Type status:** Other material. **Occurrence:** recordedBy: S.E. Thorpe; individualCount: 1; **Taxon:** scientificName: *Homotrysis macleayi* (Borchmann, 1909); **Location:** country: New Zealand; stateProvince: Auckland; verbatimLocality: Tamaki Campus (East), suburb of Saint Johns, Auckland; verbatimLatitude: 36.88100S; verbatimLongitude: 174.85310E; **Identification:** identifiedBy: Stephen E. Thorpe; **Event:** samplingProtocol: On dead tree fern frond, on ground, by pond; eventDate: 2014-01-09; **Record Level:** institutionCode: Auckland Museum; collectionCode: AMNZ87720

#### Diagnosis

*Homotrysis macleayi* is easily recognised as an alleculine tenebrionid. Fig. 3 shows the diagnostic pectinate alleculine claw. The few other alleculines present in New Zealand are all lacking a dorsal vestiture of dense setae.

#### Distribution

**Figure 1.**
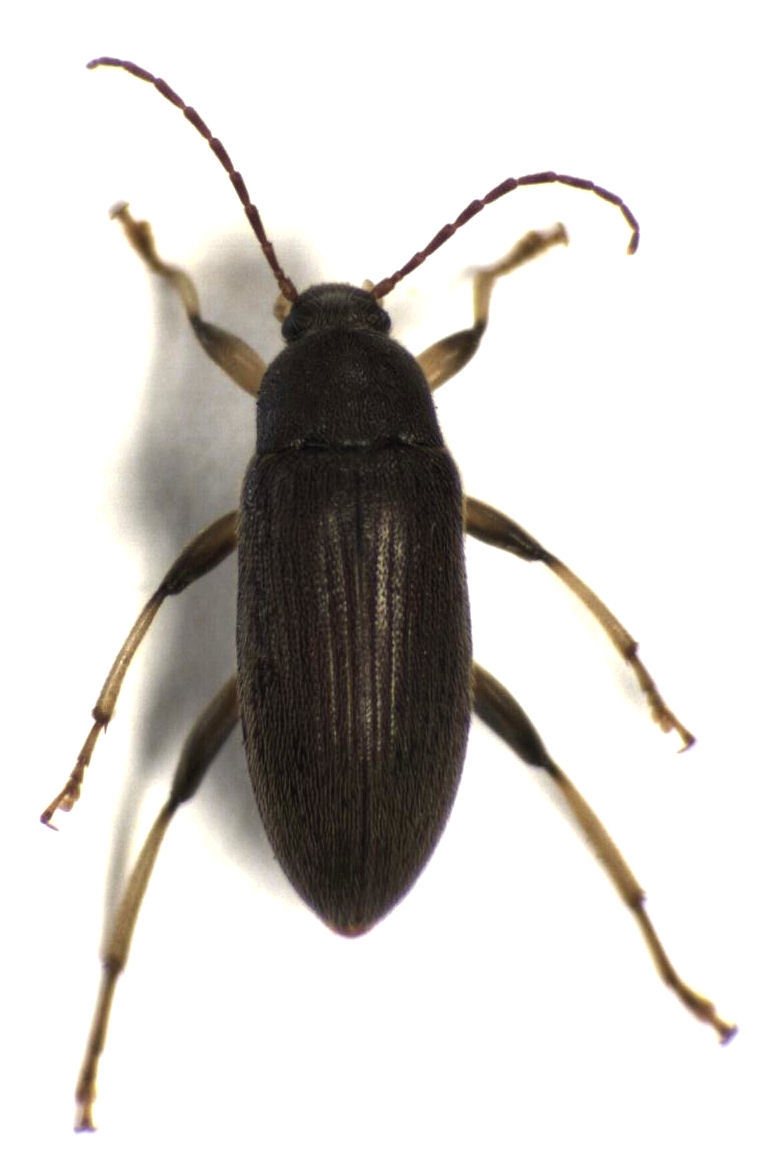
*Homotrysis macleayi*, from Tamaki Campus on 9 January 2014 (dorsal view).

**Figure 2.**
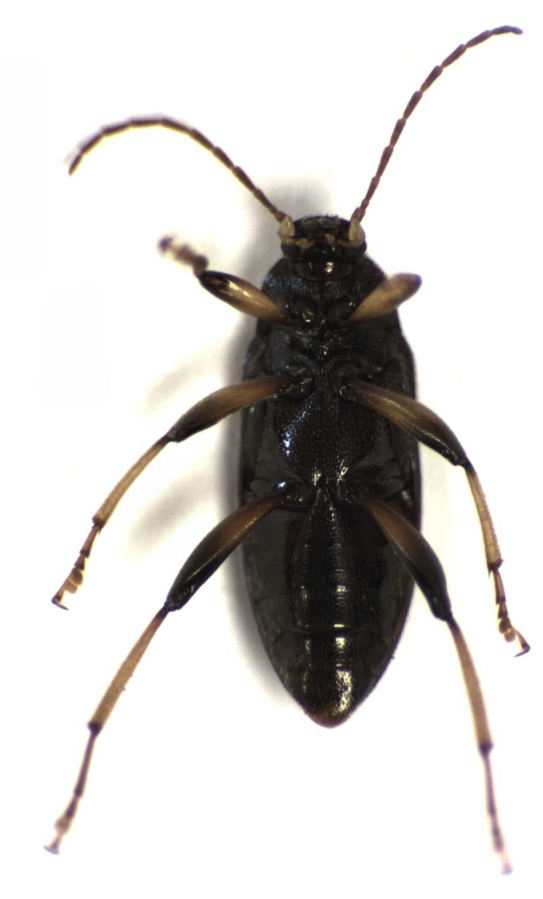
*Homotrysis macleayi*, from Tamaki Campus on 9 January 2014 (ventral view).

**Figure 3.**
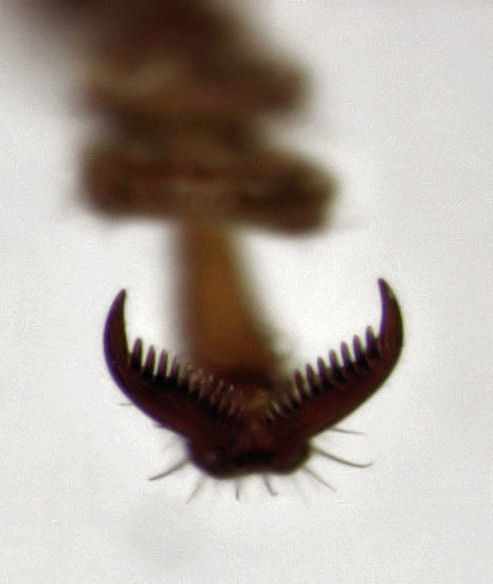
*Homotrysis macleayi*, from Tamaki Campus on 9 January 2014 (fore tarsal claw).

**Figure 4.**
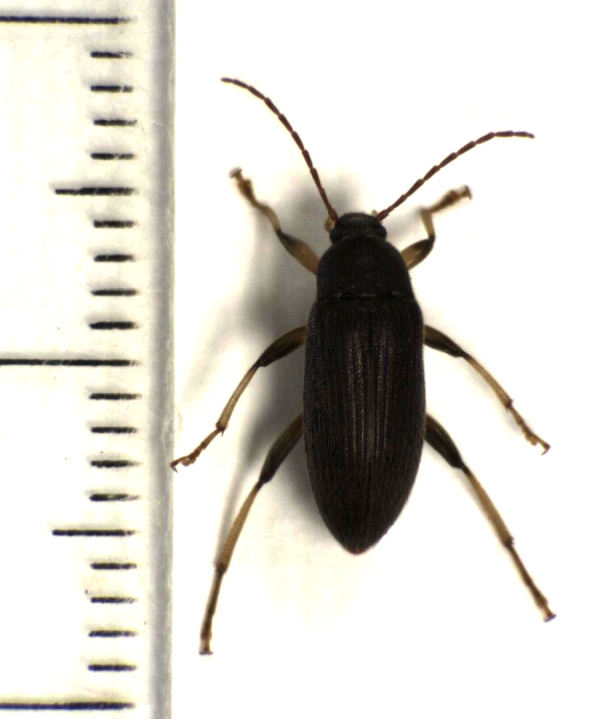
*Homotrysis macleayi*, from Tamaki Campus on 9 January 2014 (scale).

I have now collected *Homotrysis macleayi* on four separate occasions, spread over a number of years (2004, 2012, 2013, and 2014), at two sites in the vicinity of metropolitan Auckland (Auckland Domain, and the Tamaki Campus of the University of Auckland). Figs 1, 2, 3, 4 show the most recent (2014) specimen. I am not aware of any additional records from New Zealand.

#### Ecology

Little or nothing is known of the ecology of *Homotrysis macleayi*. I have collected it on a trunk of a *Eucalyptus* tree at night, under the bark of a chopped up wattle tree (by which Ministry for Primary Industries 2013 assumed that I meant *Acacia*, but which could have just as easily been brush wattle, *Paraserianthes lophantha*), under the bark of a stump of the native tree *Pittosporum eugenioides* at the edge of a carpark, and on the ground on a dead tree fern frond. I have only collected the species from anthropogenic habitats in metropolitan Auckland.

## Discussion

There is some published confusion regarding this species in Australia. *Homotrysis macleayi* was proposed as a new replacement name by Borchmann 1909 (as *Allecula macleayi*) for *Allecula flavicornis* Macleay, 1887 (Australia), which is a junior homonym of *Allecula flavicornis* Kolbe, 1883 (West Africa). It therefore makes no sense that Stone et al. 2010 in Appendix 1 (p. 314) list both *Homotrysis flavicornis* (Macleay 1887) and *Homotrysis macleayi* (Borchmann, 1909), with different associated data!

The available data clearly indicates that a breeding population of *Homotrysis macleayi* is present in the wild in metropolitan Auckland, and has been present there for some 10 years. The identification has been validated by Ministry for Primary Industries (MPI). I therefore recommend that *Homotrysis macleayi* (Borchmann, 1909) be added to the New Zealand Organisms Register (NZOR), as exotic and present in the wild.

## Supplementary Material

XML Treatment for Homotrysis macleayi
